# Editorial Comment: Are three-day voiding diaries feasible and reliable? Results from the Symptoms of Lower Urinary Tract Dysfunction Research Network (LURN) cohort

**DOI:** 10.1590/S1677-5538.IBJU.2021.04.03

**Published:** 2021-07-20

**Authors:** Jorge Moreno-Palacios

**Affiliations:** 1 IMSS Servicio Urología UMAE Hospital de Especialidades CMN Siglo XXI Ciudad de México México Servicio Urología UMAE Hospital de Especialidades CMN Siglo XXI, IMSS, Ciudad de México.

Anne P. Cameron^1^, Jonathan B. Wiseman^2^, Abigail R. Smith^2^, Robert M. Merion^2^, Brenda W. Gillespie^1^ Catherine S. Bradley^3^, et al.

Neurourol Urodyn. 2019 Nov;38(8):2185-2193

## COMMENT

Measurement of the frequency and severity of lower urinary tract symptoms (LUTS) is an important step in the evaluation and management of lower urinary tract dysfunction, voiding diaries (VD) are a semiobjective method of quantifying symptoms, they are helpful in evaluating storage symptoms, and essential in the diagnosis of nocturia. This article's aim was to describe the completeness and accuracy of a three day bladder diary in a large cohort of men and women seeking care for LUTS. Study participants were instructed to fill out a 3 day voiding and fluid intake diary. The authors divided participants into groups according to the quality of the information obtained in the VD. This cohort includes 1064 participants of which 796 (74%) returned a usable diary and 448 (56%) with complete data. Younger age was associated with a higher likelihood of not submitting a diary or submitting an unusable diary. Lower education (lower than high school) level was associated with a higher likelihood of submitting an unusable diary. Other interesting data showed that 51% of participants with complete diaries had an average 24 hour number of voids exceeding 8 (the typical threshold for defining urinary frequency), the most common missing data point was voided volume (specially in women); although in this population AUA symptom score median was 12 and about two thirds reported urgency. When comparing to questionnaires, participants tend to over represent the daytime and nighttime frequency when more than 8 or 3 times was reported respectively. The authors didn't report the prevalence of nocturnal polyuria.

This study represents one of the largest series that has analyzed the usefulness of the voiding diary. Taking into account the results, it is observed that although only half had been made correctly, useful information was achieved in 80% of the responders. A limitation in the Latin American scenario is the low educational level (1), which in this study proved to be a factor for inadequate response, so we as clinicians should take enough time to promote correct responses. However, a complete diary can be sufficient to show the voiding habits and fluid intake characteristics of our patients, and can be used to support diagnosis such as overactive bladder and polyuria (2), as well as initiate behavioral recommendations for the treatment of our patients.

Unlike other studies whose design were case-control, this is a cohort where a greater causal association can be found and the hazard ratio was calculated. In addition, this study analyzed anticholinergics used for OAB, prior research has relied largely on complex calculations using anticholinergic burden scales to account for all potential medications. However, it is a study based in prescriptions without knowing the adherence to treatment that we know is low in this disease, especially with antimuscarinics. Further research should be carried out to explore the identified effect modifiers of gender and age in this patient population, and to assess the differential effects of specific OAB anticholinergics.

The present study supports a small but measurable increased risk in dementia diagnosis with anticholinergic medications, as urologists we must be aware of this association more in the setting of elderly patients that are exposed to polypharmacy.
